# Single-cell sequencing combined with machine learning reveals the mechanism of interaction between epilepsy and stress cardiomyopathy

**DOI:** 10.3389/fimmu.2023.1078731

**Published:** 2023-01-27

**Authors:** Xuanrui Ji, Quanwei Pei, Junpei Zhang, Pengqi Lin, Bin Li, Hongpeng Yin, Jingmei Sun, Dezhan Su, Xiufen Qu, Dechun Yin

**Affiliations:** Department of Cardiology, the First Affiliated Hospital of Harbin Medical University, Harbin, China

**Keywords:** stress cardiomyopathy, epilepsy, machine learning, single-cell sequencing, stress cardiomyopathy rat model, metabolic analysis, immuno analysis

## Abstract

**Background:**

Epilepsy is a disorder that can manifest as abnormalities in neurological or physical function. Stress cardiomyopathy is closely associated with neurological stimulation. However, the mechanisms underlying the interrelationship between epilepsy and stress cardiomyopathy are unclear. This paper aims to explore the genetic features and potential molecular mechanisms shared in epilepsy and stress cardiomyopathy.

**Methods:**

By analyzing the epilepsy dataset and stress cardiomyopathy dataset separately, the intersection of the two disease co-expressed differential genes is obtained, the co-expressed differential genes reveal the biological functions, the network is constructed, and the core modules are identified to reveal the interaction mechanism, the co-expressed genes with diagnostic validity are screened by machine learning algorithms, and the co-expressed genes are validated in parallel on the epilepsy single-cell data and the stress cardiomyopathy rat model.

**Results:**

Epilepsy causes stress cardiomyopathy, and its key pathways are Complement and coagulation cascades, HIF-1 signaling pathway, its key co-expressed genes include *SPOCK2*, *CTSZ*, *HLA-DMB*, *ALDOA*, *SFRP1*, *ERBB3*. The key immune cell subpopulations localized by single-cell data are the T_cells subgroup, Microglia subgroup, Macrophage subgroup, Astrocyte subgroup, and Oligodendrocytes subgroup.

**Conclusion:**

We believe epilepsy causing stress cardiomyopathy results from a multi-gene, multi-pathway combination. We identified the core co-expressed genes (*SPOCK2*, *CTSZ*, *HLA-DMB*, *ALDOA*, *SFRP1*, *ERBB3*) and the pathways that function in them (Complement and coagulation cascades, HIF-1 signaling pathway, JAK-STAT signaling pathway), and finally localized their key cellular subgroups (T_cells subgroup, Microglia subgroup, Macrophage subgroup, Astrocyte subgroup, and Oligodendrocytes subgroup). Also, combining cell subpopulations with hypercoagulability as well as sympathetic excitation further narrowed the cell subpopulations of related functions.

## 1 Introduction

Stress cardiomyopathy (SCM) was initially considered a benign disease because of its reversible and self-limiting clinical manifestations of heart failure, but is now considered to be closely associated with the incidence of serious complications such as ventricular arrhythmias and cardiogenic shock (1). Today, the incidence of stress cardiomyopathy has been increasing due to increasing mental and social stress and increasing awareness and understanding of the disease itself (2), but its exact pathophysiological mechanisms remain unclear (3).

At present, the clinical symptoms of stress cardiomyopathy are mainly manifested as total cardiac dysfunction, retrosternal pain, elevated TNI and other symptoms similar to those of acute myocardial infarction, and its triggers are mostly related to neurological stimulation and systemic stress (4). As we all know, epilepsy is a neurological disorder that affects people of all ages and is one of the most common neurological disorders in the world, and recurrent seizures can have a persistent negative impact on the mental and cognitive functions of patients, and can even be life-threatening (5).Many studies have tried to investigate cardiac-related biomarkers under stressful stimuli (6), and epilepsy as a strong stimulus and the development of cardiovascular disease are particularly close.

The relationship between epilepsy and cardiovascular disease has been studied. For example, the extremely hypoxic environment caused by epilepsy induces elevated expression of P-gp protein in the heart and contributes to increased depolarization of cardiomyocyte membranes, ultimately leading to the development of lethal arrhythmias (7, 8). Under certain hypoxic conditions, not only the expression of P-gp protein is elevated, but also hypoxia-inducible factor-1α (HIF-1α) induces increased expression of EPO and its receptors in the brain as well as in the heart (9). These past findings will help us to decipher the mechanism of interaction between epilepsy and stress cardiomyopathy. In addition, previous studies suggest that seizures impair cardiac function, possibly through microRNA regulation (10). Such studies confirm the plausibility of a research direction to find common biomarkers between epilepsy and cardiovascular disease.

At the same time, it has been shown that the histopathological findings of heart tissue samples from patients who died suddenly and unexpectedly during epilepsy were very similar to those from patients who died during a stress cardiomyopathy episode (11), while at least one in every 1000 patients hospitalized with epilepsy had a comorbid stress cardiomyopathy according to a national study of hospitalization in epilepsy (12). Furthermore, the concept of “The Epileptic Heart” reinforces the possible link between epilepsy and stress cardiomyopathy (13) and further suggests the need for cardiac risk assessment in the clinical management of patients with epilepsy. So it is reasonable to assume that the underlying cause of death in epilepsy was most likely the occurrence of stress cardiomyopathy, but there are no studies on the common developmental mechanisms of these two diseases.

Along with the development of sequencing technology, researchers can more easily and quickly obtain the expression of a large number of genes in various diseases, which helps to understand the changes of diseases at the transcriptional level in a deeper way, and with the popularization of various high-performance machine learning algorithms in medical research (14),researchers can accurately and efficiently obtain the genes that play a key role in the development of diseases by using appropriate algorithms, which greatly advances the progress of medical research in disease mechanisms (15).

The objectives of this study were to identify pivotal genes associated with pathogenesis between stress cardiomyopathy and epilepsy and common pathogenesis and to attempt to construct a common epilepsy-stress cardiomyopathy gene regulation model. Using gene expression data from the published Gene Expression Omnibus (GEO) database (http://www.ncbi.nlm.nih.gov/geo/),we identified co-expressed genes in stress cardiomyopathy and epilepsy, while clarifying their functional status after enrichment analysis, assessing the diagnostic efficacy of candidate genes by constructing predictive models with machine learning algorithms, as well as applying single-cell data from epilepsy and constructing a rat model of stress cardiomyopathy for parallel validation. To our knowledge, this may be the first study using a systems bioinformatics approach to explore the common genetic signature between stress cardiomyopathy and epilepsy and the associated regulatory mechanisms.

## 2 Materials and methods

### 2.1 Construction of a rat model of stress cardiomyopathy

Male Sprague-Dawley rats, weighing 250 to 300 g, were selected to immobilization stress for 6 hours lasting 7 days to establish stress cardiomyopathy (SCM) model.The anesthetized rats were randomly divided into two groups of normal control and SCM. Ventricle tissues were extracted for RNA sequencing. All animal work was performed in accordance with the NIH guidelines for the use of animals in experiments.

### 2.2 Data sources

Epilepsy microarray and sequencing-related datasets selected from GSE60862 (16), GSE63808 (17), GSE143272 (18) and GSE205661 (19).GSE201048 (20) was selected for the epilepsy single cell dataset,and GSE95368 (21) was selected for the stress cardiomyopathy dataset (**Supplementary Table 1**).

### 2.3 Data preprocessing and identification of DEGs

For the epilepsy dataset, differential analysis was performed using the combination of GSE60862 (control group) and GSE63808 (disease group). Considering that the same dataset time and sequencing technology could control the batch effect to some extent (22), the limma package (23) was used to remove the batch effect while performing the differential analysis (24), and by screening (p<0.05&|logFC|>0), we obtained differential genes for up and down regulated genes in epileptic disorders. For stress cardiomyopathy, differential analysis was performed using the control and stress cardiomyopathy groups within the GSE95368 dataset, and differential genes for up-and down-regulated genes in stress cardiomyopathy were obtained by screening (p<0.05&|logFC|>0). To avoid the effect of the common batch effect in multiple datasets, the differential genes generated in the above dual diseases were intersected to obtain the dual disease co-expression differential genes.

### 2.4 Enrichment analysis to explore biological functions

The ClusterProfiler package (25) was used to perform GO enrichment analysis for dual disease homozygous upregulated genes, and the OmicShare online platform (26) was used to integrate the results of KEGG enrichment analysis and to build a network for the obtained functional pathways.

### 2.5 PPI network construction and screening of core gene modules

The PPI network was constructed for all co-expressed differential genes, the network was constructed using Cytoscape (27), and the core gene modules were searched for using MCODE (28). In contrast, the candidate genes were narrowed down using cytoHubba (29).Construction of PPI network and enrichment analysis of node genes within the network using the GENEMANIA tool (30) for the co-expressed differential genes.

### 2.6 Machine learning and rat model validation

For co-expressed differential genes, we used the epilepsy dataset GSE143272 (18), the mlr3 package (31), and tested the model performance by leaving out the cross-validation. The model selected was the Random Forest (32). On the one hand, the tree model has more relaxed requirements for input data, and on the other hand, the Random Forest technique is a classify tree technique that uses bootstrap aggregation and randomization of predictors to achieve a high degree of predictive accuracy. The above two advantages apply well to our input data. Another model choice XGBoost (33). The reason is that XGBoost has excellent performance and an efficient extreme gradient boosting based framework that can handle noise well in the data, widely promoted and used in the medical field. Then we also used XGBoost in the epilepsy dataset GSE205661 (19) for model construction and tested the model performance. Furthermore, test the model performance. Finally, we retained the candidate genes with ROC > 0.6 within the candidate genes to avoid the joint batch effect of the previous multiple datasets. By constructing the animal stress model and selecting the genes with Significant changes in both brain tissue and left ventricle (adjPvalue < 0.05 & |logFC| > 2) as the candidate genes for stress cardiomyopathy, because of the difference in sequencing depth, taking the intersection may lose more information. Conversion of rat genes to human genes by homologous mapping (34). Finally, two partial genes from the screening were extracted to obtain 21 candidate genes with up-regulated and 15 candidate genes with down-regulated expression.

### 2.7 CIBERSORT and MCPcounter immune infiltration

CIBERSORT (35) and MCPcounter (36) analyses were performed for the epilepsy dataset and the stress cardiomyopathy dataset to demonstrate immune infiltration, correlations with immune cells were calculated for candidate genes, and differences in the same immune cells were calculated by rank-sum test for different subgroups.

### 2.8 Sequencing of single cells in epilepsy combined with metabolic analysis

The Seurat package (37) was used to analyze the epilepsy single-cell dataset GSE201048 (20). Quality control criteria were set: cells with 300 to 5000 genes and mitochondrial percentage reads less than 20 were retained for subsequent analysis, while the remaining normalization, clustering, and search for differential genes were performed using standard procedures by Seurat. Principal component (PC) analysis was performed for 2000 highly variable genes, and 20 PCs were selected as input data for subsequent analysis. tSNE visualization revealed no significant batch effects across samples, so the subsequent analysis did not use algorithms such as single-cell removal of batch effects (20). Cell annotation was performed in three ways for realistic and accurate cell annotation, including manual annotation by CellMarker (38), automatic annotation by SingleR (39), and annotation based on cell surface antibody expression (40). Metabolism-related analysis was performedusing the scMetabolism package (41) to analyze relevant metabolic pathways.

## 3 Results

### 3.1 Identification of DEGs in SCM and epilepsy

**Figure 1:**
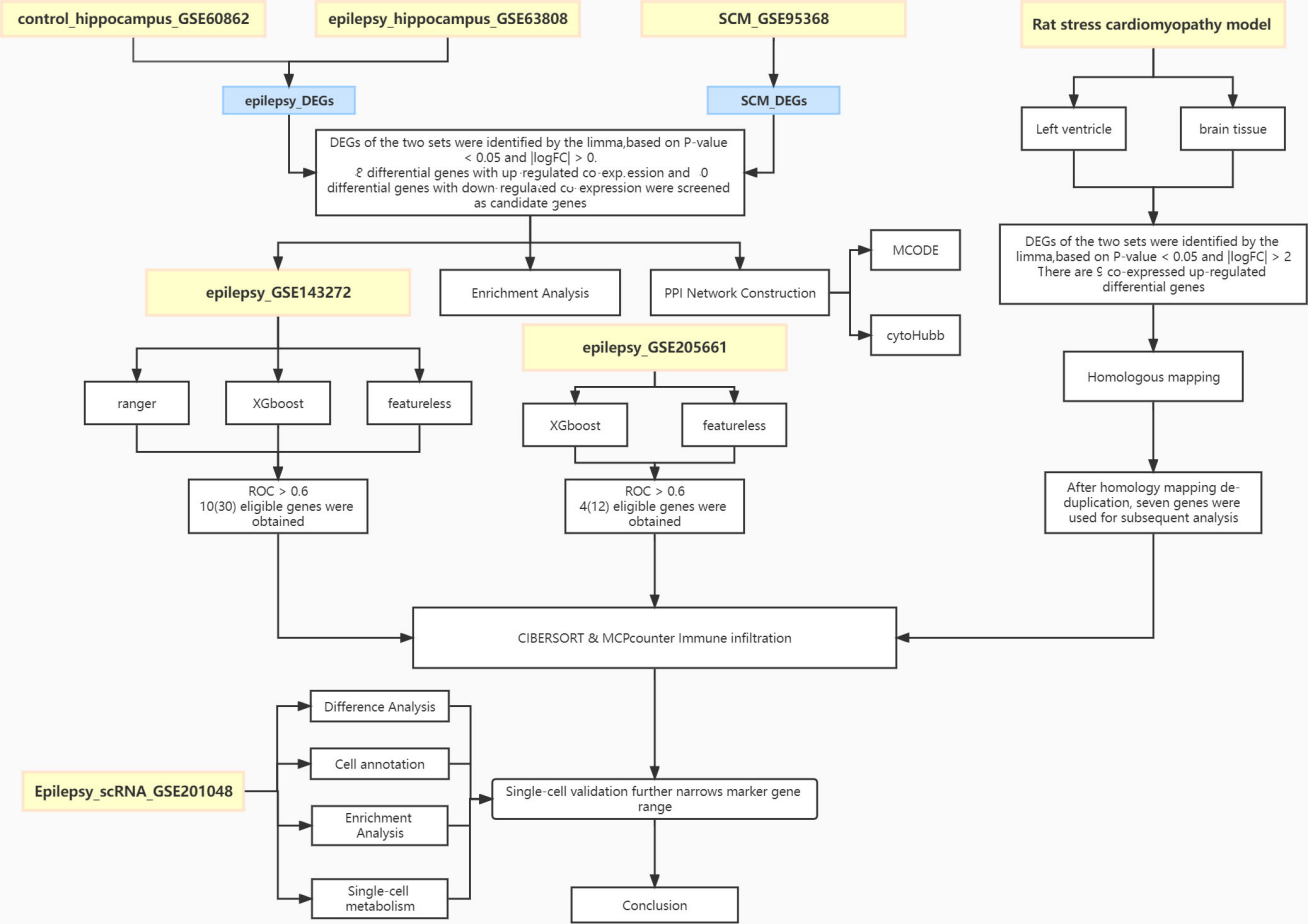
Workflow of the analysis.

**Figure 2:**
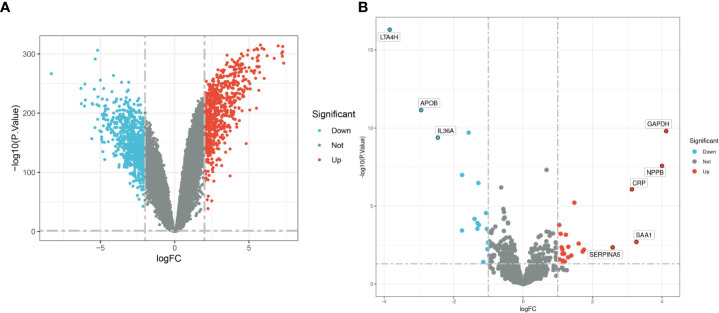
**(A)** Epilepsy-associated differential genes. **(B)** Stress cardiomyopathy DEGs.

The flow chart of this study is shown in **Figure 1**. Combined analysis of GSE60862 and GSE63808 yielded epilepsy-associated differential genes (**Figure 2A**). Differential analysis of GSE95368 yielded differential genes associated with stress cardiomyopathy (**Figure 2B**). 48 differential genes co-expressed with up-regulation and 40 differential genes co-expressed with down-regulation were obtained from the intersection of the above two parts of differential genes as the co-expressed genes for our subsequent study.

### 3.2 Co-expressed gene enrichment analysis

**Figure 3:**
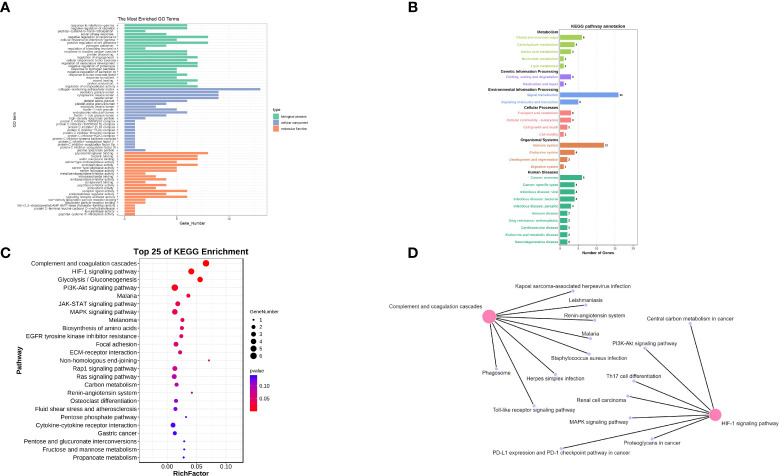
**(A)** The GO enrichment analysis of co-expressed genes. **(B)** The KEGG enrichment analysis of co-expressed genes (main class). **(C)** The KEGG enrichment analysis of co-expressed genes (pathway). **(D)** Network pathway Construction.

GO enrichment analysis was performed against 48 up-regulated co-expressed differential genes (**Figure 3A**). Specifically, the genes were mainly associated with acute-phase response, platelet alpha granule, protein C inhibitor-PLAT complex, complement binding, response to hydrogen peroxide, response to reactive oxygen species, and serine-type peptidase activity.

Subsequently, KEGG enrichment analysis was performed on 48 up-regulated co-expressed differential genes to explore the expression of the pathways, and the results were summarized and combined (**Figure 3B**). We then refined the results of the KEGG enrichment analysis (**Figure 3C**). We found that the functional alterations of the pathways were mainly in Complement and coagulation cascades, HIF-1 signaling pathway, JAK-STAT signaling pathway, PI3K-Akt signaling pathway, Rap1 signaling pathway, Ras signaling pathway, and MAPK signaling pathway. Functional alterations in these pathways provide a biological reference for refining the mechanisms of stress cardiomyopathy and epilepsy interaction. We will explore these pathways in detail in the Discussion section. Finally, we constructed a network for the pathways obtained from KEGG enrichment analysis (**Figure 3D**) and found that the central pathways were “Complement and coagulation cascades” and “HIF-1 signaling pathway”. These findings suggest that the main functions of epilepsy and stress cardiomyopathy focus on a series of functional changes caused by hypoxia and hypercoagulation.

### 3.3 PPI network construction and module analysis

**Figure 4:**
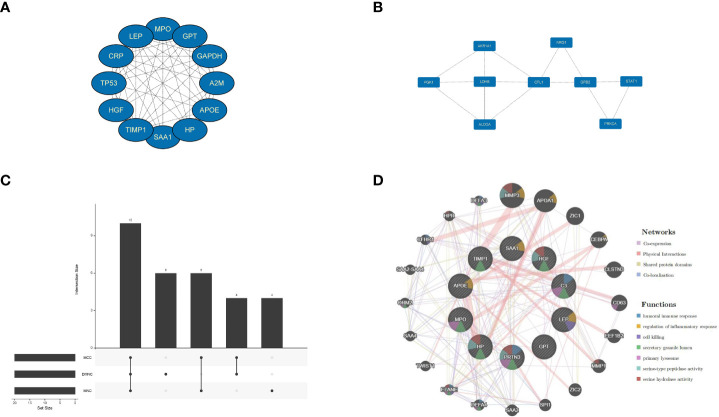
Signifificant gene module and enrichment analysis. **(A, B)** Two signifificant gene clustering modules. **(C)** CytoHubba screening of core genes (using three algorithms). **(D)** Enrichment analysis.

The PPI network was constructed for all co-expressed differential genes, and two core gene modules were obtained by MCODE (**Figures 4A, B**).To improve the accuracy, the top three algorithms with the best performance were selected for screening hub genes by cytoHubb (**Figure 4C**). Next,the PPI network was constructed for the hub gene, and the nodes were analyzed for enrichment (**Figure 4D**). By narrowing down the range of genes to focus their biological functions, we found that the functions of co-expressed genes are focused on regulation of inflammatory response, and serine-type peptidase activity. These results suggest that immune and metabolic responses are involved in disease development and will be analyzed in depth subsequently.

### 3.4 Building machine learning models to validate co-expressed genes

**Figure 5:**
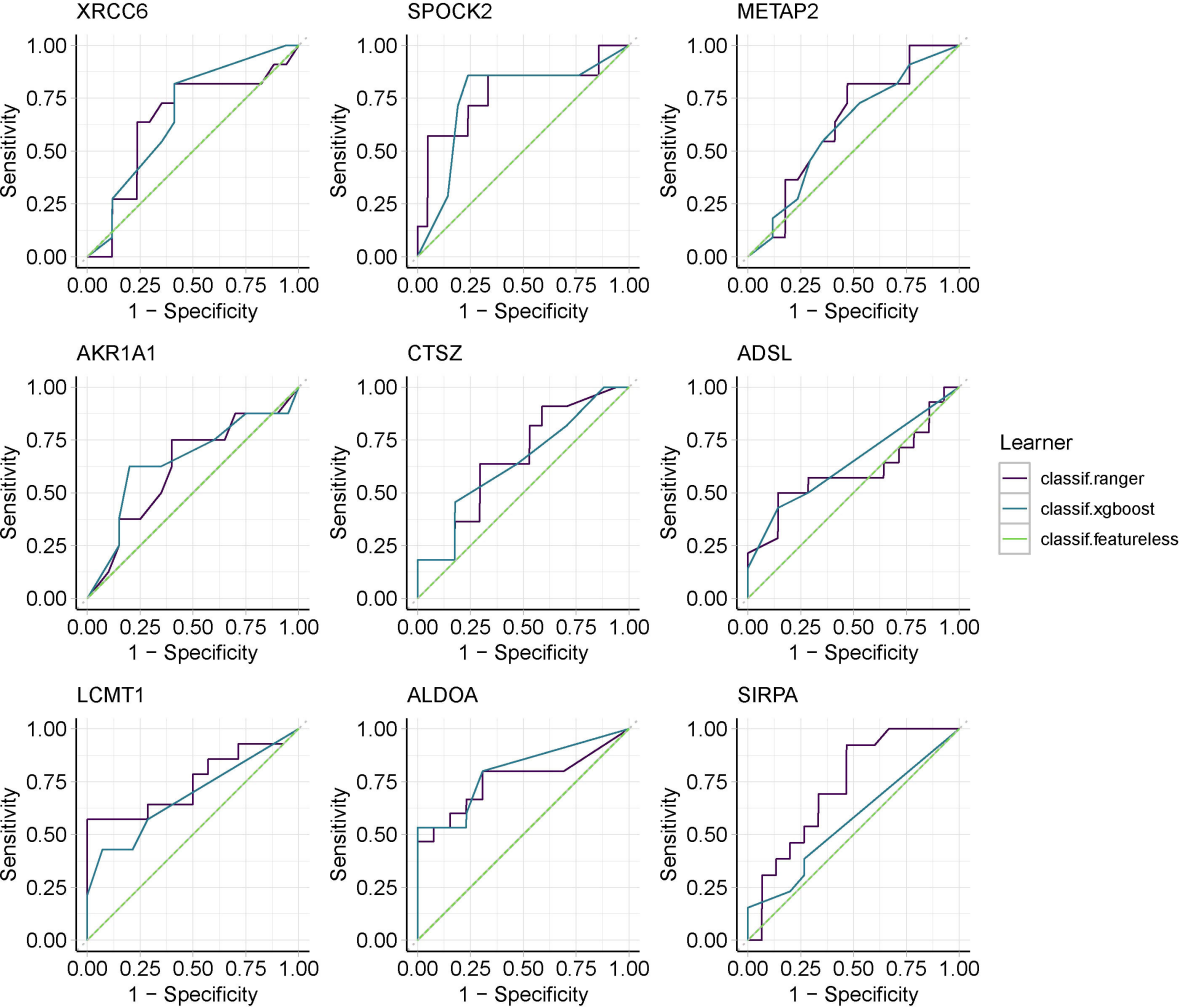
Machine learning screens for co-expressed genes with diagnostic validity,using Random Forest and XGBoost.

To verify whether co-expressed genes have diagnostic effects in epilepsy, we used independent cohorts for parallel validation, assessed the diagnostic potency of candidate genes in epilepsy by constructing random forest models and XGBoost models, and selected any model ROC greater than 0.6 as the co-expressed genes that met the conditions to be retained and visualized the results that partially met the conditions (**Figure 5** and **Supplementary Figure 1**).Then, by constructing a rat model of stress cardiomyopathy and sequencing the left ventricle and brain samples from the same sample,we finally screened for differential genes that were significantly changed in both the left ventricle and brain in stress cardiomyopathy (adjPvalue < 0.05 & |logFC| > 2), and the above two parts of the results were combined as the real dual disease co-expressed genes (**Supplementary Table 2**).

### 3.5 Immune infiltration

**Figure 6:**
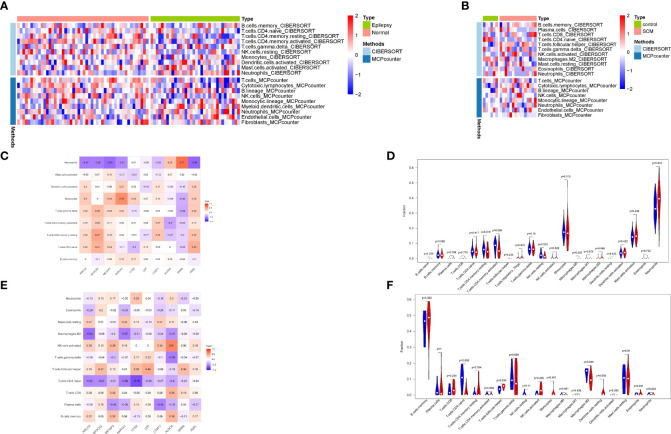
Immune infiltration analysis. **(A)** Combined analysis of CIBERSORT and MCPcounter in epilepsy. **(B)** Combined analysis of CIBERSORT and MCPcounter in SCM. **(C)** Correlation analysis of immune cells and co-expressed genes in epilepsy. **(D)** Wilcoxon rank-sum test of immune cells in epilepsy. **(E)** Correlation analysis of immune cells and co-expressed genes in SCM. **(F)** Wilcoxon rank-sum test of immune cells in SCM.

To verify the relationship between co-expressed genes and immunity,21 up-regulated co-expressed genes were subjected to immune infiltration analysis, and immune infiltration of co-expressed genes in epilepsy and stress cardiomyopathy was explored by using CIBERSORT as well as the MCPcounter algorithm. (**Figures 6A, B**).To gain a deeper understanding of the results of immune infiltration, we performed correlation analysis of immune cells with co-expressed genes for CIBERSORT and rank sum tests between immune cells under different groupings for the epilepsy dataset (**Figures 6C, D**), and similarly, we performed the same operation for the stress cardiomyopathy dataset as for the epilepsy dataset (**Figures 6E, F**).The results showed that the overall immune infiltration differed between epilepsy and stress cardiomyopathy, with an increased proportion of Neutrophils cells in epilepsy (Pvalue = 0.012) and a strong correlation between the *SIRPA* gene and Neutrophils cells (r = 0.71) and *AKR1A1* also showed a strong correlation with Monocytes cells (r = 0.59), in contrast, the proportion of T.cells and Dendritic.cells were increased in stress cardiomyopathy (Pvalue < 0.05), and some genes, such as the *LEP* gene were moderately correlated with T.cells (r = 0.45), *ALDOA* gene was highly correlated with NK.cells (r = 0.61). The above results suggest that the immune mechanisms of the two diseases are not identical, with an increased proportion of neutrophils in epilepsy leading to the release of inflammatory mediators, which in turn can be pro-epileptic factors, and conversely, an increased proportion of T cells in stress cardiomyopathy suggesting that the duration of the disease is not as transient as previously perceived, while obtaining immune-related genes in the respective diseases, the function of which will be the focus of subsequent discussion.

### 3.6 Epilepsy single-cell sequencing

**Figure 7:**
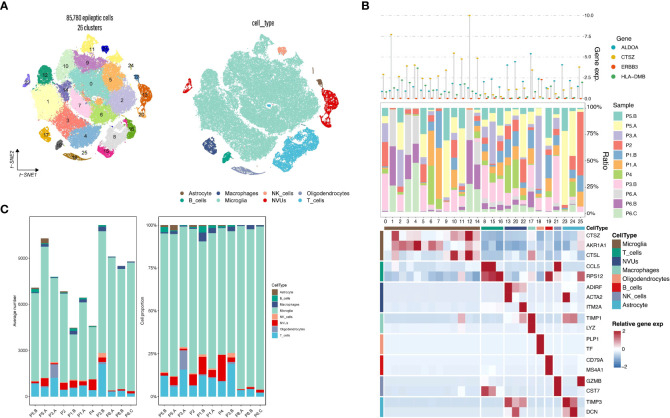
Epilepsy single-cell analysis. **(A)** Single-cell subpopulation clustering and cell annotation. **(B)** Three-layered complex heatmap of selected co-expressed genes in each cell cluster. Top: Mean expression of co-expressed markers. Middle: Tissue preference of each cluster; Bottom: Relative expression map of known marker genes associated with each cell subset. Mean expression values are scaled by mean-centering, and transformed to a scale from -2 to 2. **(C)** Average cell number and relative proportion of subsets from tissues of each origin.

In our preliminary hypothesis, epilepsy plays a “trigger” role in both diseases. To improve our understanding of the immune mechanism of epilepsy, we performed analysis using the epilepsy single-cell dataset, which was derived from six samples with epilepsy single-cell dataset contains 85,780 cells. We obtained a downscaled clustering map by performing the standard procedure of single-cell analysis to generate a total of 26 subgroups. We then annotated the cell subgroups by combining CellMarker database annotation, SingleR annotation, and cell surface antibody annotation, resulting in 8 functional subgroups (**Figure 7A**). Most single cell suspensions obtained from epileptic brain tissue were annotated with known cell types, including Microglia, T_cells, NVUs, Macrophages, etc (**Figure 7B**). It can be observed that most of the cell subpopulations were defined as Microglia. By contrast, one distinct cluster was identified as oligodendrocytes in the P1.B, P3.A, and P3.B samples. At the same time, immune cell infiltration is observed in epilepsy samples originating from different individuals and brain regions (**Figure 7C**). Validation of epilepsy and stress cardiomyopathy co-expressed genes at the single-cell level revealed that *ALDOA*, *CTSZ*, *ERBBS*, *HLA-DMB* and other genes were validated as double disease significant co-expression genes. As a result of the previous analysis, some genes showed a significant correlation with the proportion of immune cells among the co-expressed genes validated at the single-cell level. Suggesting that immunity may act as a bridge in epilepsy and stress cardiomyopathy functions of these genes and the subgroups they belong will be explored in subsequent discussions.

### 3.7 Metabolic analysis

**Figure 8:**
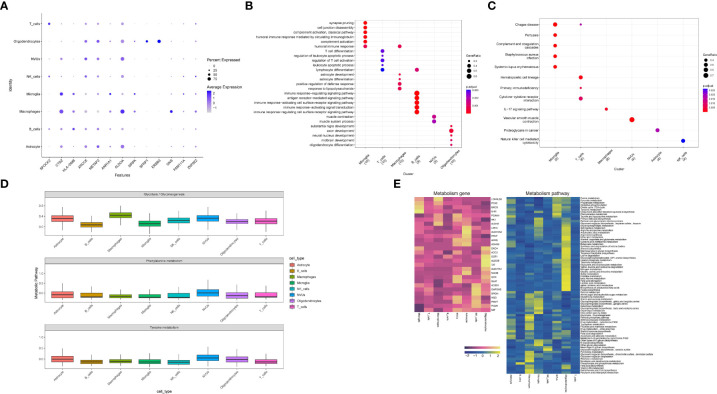
Functional enrichment analysis and metabolic analysis at the level of single-cell subpopulations. **(A)** Expression of co-expressed genes on functional subgroups. **(B, C)** Enrichment analysis of single cell subpopulations. **(D)** Metabolic pathway expression quantification for single-cell subpopulations. **(E)** Quantification of metabolic pathways and metabolism-related gene expression.

Co-expressed genes were validated at the single cell level, first considering the significance (Pvalue < 0.05) and showing the expression changes of co-expressed genes in eight functional subgroups (**Figure 8A**), including *SPOCK2*, *CTSZ*, *HLA-DMB*, *XRCC6*, *METAP2*, *AKR1A1*, *ALDOA*, *SFRP1*, *ERBB3*, *GNS*, *ZNF622*. The eight single-cell functional subpopulations were enriched separately to identify the functional status of each subpopulation (**Figures 8B, C**). The enrichment analysis results strongly suggest T-lymphocyte and neuroglial activation, such as regulation of T cell activation, T cell differentiation, astrocyte differentiation, immune response-regulating signaling pathway, response to lipopolysaccharide and astrocyte development. The emerging term “autoimmune epilepsy” highlights the role of the immune system and its dysregulation in epileptogenesis. Our results suggest that the occurrence of epilepsy is the result of a combination of immune mechanisms and structural alterations.

On the other hand, the enrichment analysis results laterally verified our cellular annotation accuracy.In addition, enrichment analysis results for pathways suggest alterations in the immune system, such as the IL-17 signaling pathway, Natural killer cell mediated cytotoxicity, and alterations in the coagulation system (Complement and coagulation cascades) play a role in the progression of the disease. The follow-up will be an integrated exploration of the two diseases.

From our previous work, we know that the release of catecholamine hormone in large amounts may be one of the potential mechanisms in the pathogenesis of stress cardiomyopathy,we considered that the release of catecholamines might also be one of the common pathogenic mechanisms in epilepsy and stress cardiomyopathy, so we performed metabolic correlation analysis on single cell functional subpopulations (**Figure 8D**). By selecting three metabolic pathways, Glycolysis/Gluconeogenesis, Phenylalanine metabolism and Tyrosine metabolism, and then evaluating the functional subgroups. The results suggest that NVUs, Macrophage and Oligodendrocytes may be the focus of our subsequent studies on metabolism-related changes. Finally, we demonstrated the metabolism-related pathways and metabolism-related genes at the single-cell level (**Figure 8E**).

Finally, the subpopulations to which the screened co-expressed genes belonged were mainly T_cells, NVUs, Microglia, Oligodendrocytes, Astrocyte, Macrophage, and combined with the results of metabolic analysis, NVUs, Macrophage and Oligodendrocytes were finally identified. Our findings identified and obtained the genes that play a major role in epilepsy and stress cardiomyopathy and the Biological functions of disease development and also refined the results to obtain the cellular subpopulation to which the gene belongs (**Supplementary Table 3**).

## 4 Discussion

This study is the first to focus on the common disease mechanisms between epilepsy and stress cardiomyopathy. A review of previous literature found that the cardiac pathology of patients with sudden death in epilepsy is very similar to that of stress cardiomyopathy (11). The brain-center interaction mechanism is also widely recognized in stress cardiomyopathy (42). These findings inspired us to try to find a common pathogenic mechanism between epilepsy and stress cardiomyopathy.

By merging two large epilepsy datasets and performing differential analysis, we believe that excessive removal of batch effects by the algorithm may remove most of the biological differences (43), so here we use the built-in function of the limma package to remove batch effects and take the intersection with the results of the stress cardiomyopathy differential analysis to try to circumvent some of the batch effects. For the genes generated by the intersection, we define them as co-expressed genes in epilepsy and stress cardiomyopathy (48 genes up-regulated,40 genes down-regulated).

To clarify the common biological functions of epilepsy and stress cardiomyopathy, an Enrichment analysis was performed. GO enrichment analysis showed the genes were mainly associated with acute-phase response, platelet alpha granule, protein C inhibitor-PLAT complex, complement binding, response to hydrogen peroxide, response to reactive oxygen species, and serine-type peptidase activity (**Figure 3A**). In our preliminary hypothesis, epilepsy as a strong stressful stimulus leads to the development of stress cardiomyopathy. In cardiac tissue, hyperactivity of the coagulation system leads to left ventricle thrombus formation (4), and the clinical manifestations of stress cardiomyopathy confirm the correctness of our conclusion that there is coagulation system activation in the development of stress cardiomyopathy due to epilepsy.

Moreover, the hyperactivity of the immune system can be elaborated separately from the heart and the brain, in the heart mainly shows the activation of the immune system, and the later analysis will focus on exploring. In brain tissue, the relationship between epilepsy and immunity has been studied (44), and existing findings prefer immunity as a component of epilepsy pathogenesis. Subsequent analyses will explore changes in cell subpopulations through single-cell-level data.

Finally, the appearance of oxidative stress function suggests, on the one hand, the existence of a hypoxic environment in the interaction mechanism of epilepsy leading to stress cardiomyopathy. On the other hand, oxidative stress can damage endothelial cells and lead to abnormal cardiac function. Our results strongly point to epilepsy causing stress cardiomyopathy being plausible from a biological functional point of view. In contrast, the triad “hypoxia - oxidative stress - inflammation” is a vicious circle where anyone can initiate the activation of others (45). At the same time, the hypercoagulation system contributes to the formation and aggravation of the hypoxic environment of the body.

KEGG enrichment analysis revealed that the pathway alterations were mainly concentrated in Complement and coagulation cascades, HIF-1 signaling pathway, JAK-STAT signaling pathway, PI3K-Akt signaling pathway, Rap1 signaling pathway, Ras signaling pathway, and MAPK signaling pathway. The core pathway is the Complement and coagulation cascades and the HIF-1 signaling pathway (**Figures 3B–D**).

We have identified blood hypercoagulability as one of the functional alterations in stress cardiomyopathy due to epilepsy (46). Meanwhile, combined with the hypoxic environment leading to increased expression of hypoxia-inducible factor 1α (HIF-1α), as well as the clinical symptoms of stress cardiomyopathy and previous studies, we suggest that hypoxia-inducible factor induces elevated P-gp protein expression in the hypoxic environment caused by epilepsy, and high expression in the brain may then lead to the development of drug resistance, while high expression in the heart leads to increased myocardial cell membrane depolarization, resulting in lethal the development of arrhythmias (7), which is consistent with the complications of stress cardiomyopathy, and this acute hypoxic environment also promotes cardiac EPO/EPO-R expression, which protects the myocardium to some extent (9).

We found activation of the JAK/STAT signaling pathway during epilepsy-induced stress cardiomyopathy, a pathway thought to be associated with the stress response, and to date, various vascular stressors have been linked to this pathway, including angiotensin II effects, oxidative stress, and immune responses (47), which combined with the clinical manifestations of stress cardiomyopathy, lead us to believe that the JAK/STAT signaling pathway reasonable view is that epilepsy-induced stress stimuli create a hypoxic environment that activates hypoxia-inducible factor 1α (HIF-1α) and upregulates their associated pathways and induces activation of the JAK/STAT signaling pathway thereby mediating the regulation of cardiovascular smooth muscle cells and endothelial cells by angiotensin II and oxidative stress as well as the neuroprotective function of EPO (48).

However, We suggest that homeostatic mechanisms exist in the organism even under extreme conditions, and the Epac2-Rap1 signaling pathway antagonizes the effects of oxidative stress on cardiovascular endothelial cells (49). In contrast, activation of the Ras signaling pathway may exacerbate the hypoxic environment in the brain and heart, potentially pushing the above balance toward an imbalance. In contrast, activation of the Ras signaling pathway (50) and MAPK signaling pathway (51) may promote an increased hypoxic environment in the brain and heart as well as an increased level of oxidative stress, potentially tipping the balance towards an imbalance.

Constructing a core module for PPI network discovery will give us a preliminary shaping of the molecular interaction mechanism between epilepsy and stress cardiomyopathy (**Figures 4A, B**). Next, we used cytoHubb to narrow down the gene range and selected three algorithms that have been demonstrated to perform better in the literature for screening genes (29) (**Figure 4C**). The enrichment analysis of the nodal genes in the PPI network allowed further insight into the biological functions hidden by numerous genes, and inflammatory immune mechanisms were found in the interaction between epilepsy and stress cardiomyopathy, suggesting that immunity may play a “maintenance” function in both diseases. This part of the results will be discussed in conjunction with the subsequent immune infiltration analysis (**Figure 4D**).

In previous studies, serine-type peptidase activity has been associated with coagulation and complement activation (52). It is interesting to note that coagulation has been repeatedly implicated in the functional mechanisms of epilepsy and stress cardiomyopathy.We believe that in patients with coronary artery disease, epilepsy leading to stress cardiomyopathy may be a stimulating factor that leads to further damage to the originally abnormal coronary arteries and the occurrence of acute coronary syndrome. So patients with coronary artery abnormalities do not exclude the possibility of stress cardiomyopathy, and even stress cardiomyopathy is a contributing factor to the acute coronary syndrome (46). Meanwhile, the relationship between stroke and epilepsy has been studied (53), so it is reasonable to assume that epilepsy leads to stress cardiomyopathy and increases the frequency and duration of epilepsy through a combination of abnormal coagulation mechanisms and hemodynamic disturbances resulting in further cerebrovascular damage.

Because of the popularity of machine learning in clinical research and its high performance (15), we chose the integrated learning model random forest and the extreme gradient boosting XGBoost, which has good performance in clinical diagnosis. Because of the mismatch in the number of genes due to the different sequencing depths of different datasets, we selected two datasets for model construction. We constructed the model using a cross-validation method. Selected genes with ROC greater than 0.6 are to be retained (**Figure 5**).

Considering the batch effect and sequencing depth, we constructed a rat model of stress cardiomyopathy and sequenced the brain and left ventricle separately. In our previous work, there is a theory of heart-brain interaction in stress cardiomyopathy (1). This supports our combined analysis and reasonable for constructing a model of stress cardiomyopathy to sequence both brain and heart samples. We retained the overexpressed genes in the brain and heart (adjPvalue < 0.05 & |logFC| > 2) and validated the genes detected in stress cardiomyopathy. Homologous mapping of genes by biomart. By combining the above two parts, we further screened and finally obtained 21 candidate genes with up-regulated and 15 candidate genes with down-regulated expression.

Considering the involvement of immune mechanisms in the common biological functions of epilepsy and stress cardiomyopathy, immune infiltration analysis was performed separately for epilepsy and stress cardiomyopathy, and the correlation between the ratio of co-expressed genes and immune cells was performed (**Figure 6**). The available data shows that the immune response in the heart and brain tissue is not the same. We believe that immunity is a systemic response, so it is likely that the immune response acts as another bridge between epilepsy and stress cardiomyopathy. According to our results, in epilepsy-induced stress cardiomyopathy, the proportion of neutrophils in the brain tissue increases and releases inflammatory mediators leading to the further persistence of epilepsy (54). This step is initiated by establishing a hypoxic environment and elevated expression of HIF-1α. At the same time, immune inflammation in the brain causes sympathetic excitation, affecting cardiac function *via* the nervous system (55).

At the same time, in the heart, the increased proportion of monocytes and the imaging findings in stress cardiomyopathy demonstrate that systemic inflammation may play a key role in the pathology of the disease (56). We observed a decrease in the proportion of M2 macrophages, suggesting that inflammatory mechanisms do not play a transient role in the disease (57). As previously perceived and that as inflammatory mechanisms act in the heart, the effects on cardiac ejection function lead to a further exacerbation of the hypoxic environment in the organism, which ultimately leads to a further exacerbation of immune inflammation as well as oxidative stress in the brain.

By correlating co-expressed genes as well as immune cell ratios, we obtained genes with significant correlation with immune cell ratios (*SIRPA*, *AKR1A1*, *LEP*, *ALDOA*) that will be validated in epilepsy data at the single cell level in an attempt to find immune genes that link heart and brain tissue.

Next, we tried to obtain a more comprehensive biological interpretation by enhancing the sequencing depth. We used the epileptic single-cell dataset to get a more comprehensive understanding of our candidate genes. Through the standard Seurat process, we clustered 85780 epileptic cells into 26 subgroups (**Figure 7A**). Cellular annotation is a difficult part of single-cell analysis, and this step is highly subjective. We used three dimensions of information to annotate the cells comprehensively. On the one hand, we obtained the top ten differential genes for each subpopulation by differential analysis of 26 subpopulations. We manually annotated them by combining the differential genes with the Cellmarker database. On the other hand, we used SingleR for automatic annotation. Although the accuracy of SingleR annotation is not as good as that of manual annotation, it provides us with references and helps to select the results from Cellmarker annotation. The last aspect is that we selected CITE-seq technology, which is a technique to obtain both intracellular gene expression and protein expression, CD45 protein expression level can distinguish immune cells from non-immune cells, CD19 and CD20 protein expression levels can mark B cells, CD14 can help us to mark macrophages, and finally, we mark neurovascular unit (NVU) for non-immune cells by marker genes (20).

We visualized the expression of some co-expressed genes in different subpopulations (**Figure 7B**). We found that the expression of candidate genes in different subpopulations is distinct, which also tells us that only some cell subpopulations may be involved in the pathogenesis of dual diseases. We also validated the co-expressed genes at the single-cell level. Considering that a hard threshold may hurt the results, we considered two sets of screening metrics separately: on the one hand, we limited the candidate genes to meet Pvalue < 0.05 in the single-cell data set, so we obtained 12 co-expressed genes, and on the other hand, we limited the candidate genes to meet Pvalue < 0.05 and |logFC| > 1 in the single-cell data set, so that we obtained 6 candidate genes. We considered the relationship between pvalue, logFC and pct. We considered logFC as the most important reference in single-cell sequencing, so in defining the subpopulation to which the candidate gene belongs, we selected the subpopulation to which the candidate gene belongs with pvalue < 0.05 and |logFC| >1, and if there are multiple subpopulations, we considered the subpopulation to which the candidate gene belongs with pct > 0.5. In this way, we successfully narrowed down the candidate genes and localized the specific functional subgroups to which they belonged (**Supplementary Table 3**).

Thus, we obtained the co-expressed genes of epilepsy and stress cardiomyopathy and the cellular subgroups they belong to in brain tissue, including the co-expressed up-regulated genes (*SPOCK2* in the T_cells subgroup, *CTSZ* in the Microglia subgroup, *HLA-DMB* in the Microglia subgroup, *ALDOA* was expressed in NVUs and Macrophage and Astrocyte subgroups, *SFRP1* in the Oligodendrocytes subgroup, *ERBB3* in the Oligodendrocytes subgroup) and the co-expressed down-regulated genes (*PRKCA* in the Macrophages subgroup, *C3* in the Microglia subgroup, *GSTM3* in the Astrocyte subgroup).

According to the previous results, in stress cardiomyopathy, the *ALDOA* gene is positively associated with NK cells but negatively associated with M2 macrophages. At the same time, in epileptic brain tissue, it is mainly expressed in macrophages and astrocytes. It has been demonstrated that the *ALDOA* gene is associated with macrophage and T lymphocyte infiltration (58), while astrocytes are also associated with central nervous system inflammation (59). Our results allow us to hypothesize that the *ALDOA* gene is involved in neuroinflammation through astrocytes and macrophages, affecting sympathetic and metabolic activities through neuroinflammation. Meanwhile, high *ALDOA* expression in the heart is accompanied by a decrease in the proportion of M2 macrophages, leading to an inflammatory manifestation with extensive infiltration in the heart, which is consistent with clinical manifestations of stress cardiomyopathy are consistent.

Taking together our results and previous literature, we found that *SFRP1* protects cardiomyocytes in the heart by inhibiting the Wnt signaling pathway (60). The regulation of *SFRP1* in epileptic disorders has also recently started to receive attention (61). However, there are still relatively few relevant studies, and our results could help to target the cellular subpopulation of *SFRP1* in epilepsy and refine the antagonistic role of *SFRP1* in epilepsy.

Previous studies have found that *ERBB3* expression in the heart is associated with an adaptive response to stress, which is identical to our results (62), and studies in the brain with *ERBB3* and Obstructive sleep apnea syndrome (OSAS), which also shaped the hypoxic environment and confirmed that *ERBB3* was involved in and reduced the hypoxia-induced inflammatory response (63), but *ERBB3* has not yet been studied in epilepsy, which will be the direction of our subsequent studies. No studies in the same field have confirmed the role of *SPOCK2*, *CTSZ* and *HLA-DMB* in epilepsy or stress cardiomyopathy. However, it has been demonstrated that *SPOCK2* acts as a susceptibility gene for bronchial dysplasia (64) and exacerbates hyperoxia-related lung injury (65). Our results provide directions for subsequent studies on the interaction mechanism between epilepsy and stress cardiomyopathy.

Our results found that *C3* expression is reduced in the interaction between epilepsy and stress cardiomyopathy. It has been shown that blocking *C3* protects against neuronal damage in Although Alzheimer’s disease (AD) (66). Interestingly, *C3* activates the JAK2/STAT3 pathway and is associated with the progression of gastric cancer (67), these two patterns of regulation of different pathways of the same gene in different tissues give us a hint that reduced *C3* gene expression in Epilepsy and stress cardiomyopathy may have an important role. We conclude that *C3* reduction protects neurons from damage during stressful conditions.

Next, we performed enrichment analysis by cell subpopulation, and the GO enrichment analysis results showed a high consistency between subpopulation and function, which also illustrates the accuracy of the previous cell annotation (**Figure 8B**). The results of GO enrichment analysis were T cell activation, response to lipopolysaccharide, and astrocyte development. Firstly, T cell activation suggests that in the interaction between epilepsy and stress cardiomyopathy, lymphocytes are predominant in the brain (44). We believe that epilepsy acts as a “trigger” in both diseases. Hence, it suggests that T-cell targeted therapy is promising in the co-development of both diseases. Our results also suggest that epilepsy is a product of immune mechanisms and organic changes (44).

Secondly, the response to lipopolysaccharide is compared to our previous results, where we found reduced expression of *PRKCA* in epilepsy and stress cardiomyopathy. *PRKCA* was associated with lipopolysaccharide (LPS) induced neuroinflammatory response (68), and *PRKCA* in the brain is localized to the macrophage subpopulation. It has been found that the protective mechanism of *PRKCA* is achieved by inhibiting the release of pro-inflammatory cytokines through macrophages and the MAPK signaling pathway (69). Our results suggest that this protective mechanism may be inhibited in both disease interactions.

Finally, we performed KEGG enrichment analysis showing alterations in the IL-17 signaling pathway, Natural killer cell mediated cytotoxicity, and Complement and coagulation cascades, among which Microglia cell subpopulation, as well as Macrophages cell subpopulation, may play important functions in epilepsy causing stress cardiomyopathy according to our results (**Figure 8C**).

Meanwhile, in order to explore the remaining possible mechanisms of epilepsy causing stress cardiomyopathy, we conducted metabolic-related analyses (**Figures 8D, E**), and based on our preliminary results, we believe that sympathetic excitation is a more plausible explanation for the mechanisms by which the brain affects the heart, so we selected three metabolic pathways for analysis based on this, and eventually targeted three cell subpopulations in the brain, namely, NVUs, Macrophage, and Oligodendrocytes. Moreover, we went on to try to expand our results to obtain highly metabolic genes with corresponding cell subpopulations (*GOT1*, *ALDOB*, *TAT*, *LDHAL6B*).

In conclusion, our work proposes that epilepsy causes stress cardiomyopathy and explores a possible common mechanism for this dual disease for the first time. There are two major functional pathways, including the Complement and coagulation cascades and the HIF-1 signaling pathway. We believe that the two functional alterations, coagulation, and hypoxia, affect each other, but hypoxia is the initiating signal. This is followed by a “hypoxia - oxidative stress - inflammation” in the brain, where there are pathways that act as stimulators and inhibitors, followed by immune mechanisms in the brain and heart that are elucidated in epilepsy-induced stress cardiomyopathy. Finally, the localization of co-expressed genes at the single-cell level. I believe our work will contribute to the study of stress cardiomyopathy due to epilepsy and the study of brain-heart interaction.

## Data availability statement

The original contributions presented in the study are included in the article/**Supplementary Material**. The data presented in the study are deposited in the GEO repository, accession number GSE223385.

## Ethics statement

All animals were kept in a pathogen-free environment and fed ad lib. The procedures for care and use of animals were approved by the Ethics Committee of the First Affiliated Hospital of Harbin Medical University and all applicable institutional and governmental regulations concerning the ethical use of animals were followed.

## Author contributions

XJ: Conceptualization (equal); data curation (equal); writing – original draft (lead). QP and JZ: methodology (equal); project administration (equal); Formal analysis (equal); writing – original draft (equal). PL and BL: Investigation (equal); project administration (equal). HY, JS, and DS: Investigation (equal); project administration (equal). XQ: Conceptualization (equal); supervision (equal); validation (equal); DY: Investigation (lead); project administration (lead); writing – original draft (lead); writing – review and editing (lead). All authors contributed to the article and approved the submitted version.

## References

[1] Medina de Chazal H Del Buono MG Keyser-Marcus L Ma L Moeller FG Berrocal D. Stress cardiomyopathy diagnosis and treatment: JACC state-of-the-Art review. J Am Coll Cardiol (2018) 72(16):1955–71. doi: 10.1016/j.jacc.2018.07.072 PMC7058348 30309474

[2] Tu T Li J Fang Z Hu X Tang J Zhao Y. In-hospital cardiac arrest after emotional stress in a patient hospitalized with gastrointestinal symptoms and chronic anxiety disorder. Cardiovasc Innov Appl (2021) 6(1):57–61. doi: 10.15212/CVIA.2021.0021

[3] Dawson DK. Acute stress-induced (takotsubo) cardiomyopathy. Heart (British Cardiac Society) (2018) 104(2):96–102. doi: 10.1136/heartjnl-2017-311579 28824005

[4] Zhang L Piña IL. Stress-induced cardiomyopathy. Heart Failure Clinics (2019) 15(1):41–53. doi: 10.1016/j.hfc.2018.08.005 30449379

[5] Thijs RD Surges R O'Brien TJ Sander JW. Epilepsy in adults. Lancet (London England) (2019) 393(10172):689–701. doi: 10.1016/S0140-6736(18)32596-0 30686584

[6] Nass RD Motloch LJ Paar V Lichtenauer M Baumann J Zur B. Blood markers of cardiac stress after generalized convulsive seizures. Epilepsia (2019) 60(2):201–10. doi: 10.1111/epi.14637 30645779

[7] Auzmendi J Buchholz B Salguero J Cañellas C Kelly J Men P. Pilocarpine-induced status epilepticus is associated with p-glycoprotein induction in cardiomyocytes, electrocardiographic changes, and sudden death. Pharm (Basel Switzerland) (2018) 11(1):21. doi: 10.3390/ph11010021 PMC5874717 29462915

[8] Auzmendi J Akyuz E Lazarowski A. The role of p-glycoprotein (P-gp) and inwardly rectifying potassium (Kir) channels in sudden unexpected death in epilepsy (SUDEP). Epilepsy Behav E&B (2021) 121(Pt B):106590. doi: 10.1016/j.yebeh.2019.106590 31706919

[9] Auzmendi J Puchulu MB Rodríguez JCG Balaszczuk AM Lazarowski A Merelli A. EPO and EPO-receptor system as potential actionable mechanism for the protection of brain and heart in refractory epilepsy and SUDEP. Curr Pharm design (2020) 26(12):1356–64. doi: 10.2174/1381612826666200219095548 32072891

[10] Pansani AP Ghazale PP Dos Santos EG Dos Santos Borges K Gomes KP Lacerda IS. The number and periodicity of seizures induce cardiac remodeling and changes in micro-RNA expression in rats submitted to electric amygdala kindling model of epilepsy. Epilepsy Behav E&B (2021) 116:107784. doi: 10.1016/j.yebeh.2021.107784 33548915

[11] Templin C Ghadri JR Diekmann J Napp LC Bataiosu DR Jaguszewski M. Clinical features and outcomes of takotsubo (Stress) cardiomyopathy. New Engl J Med (2015) 373(10):929–38. doi: 10.1056/NEJMoa1406761 26332547

[12] Desai R Singh S Patel U Fong HK Kaur VP Varma Y. Frequency of takotsubo cardiomyopathy in epilepsy-related hospitalizations among adults and its impact on in-hospital outcomes: A national standpoint. Int J Cardiol (2020) 299:67–70. doi: 10.1016/j.ijcard.2019.07.034 31327513

[13] Verrier RL Pang TD Nearing BD Schachter SC. The epileptic heart: Concept and clinical evidence. Epilepsy Behav E&B (2020) 105:106946. doi: 10.1016/j.yebeh.2020.106946 32109857

[14] Deo RC. Machine learning in medicine. Circulation (2015) 132(20):1920–30. doi: 10.1161/CIRCULATIONAHA.115.001593 PMC5831252 26572668

[15] Johnson KW Torres Soto J Glicksberg BS Shameer K Miotto R Ali M. Artificial intelligence in cardiology. J Am Coll Cardiol (2018) 71(23):2668–79. doi: 10.1016/j.jacc.2018.03.521 29880128

[16] Trabzuni D Ramasamy A Imran S Walker R Smith C Weale ME. Widespread sex differences in gene expression and splicing in the adult human brain. Nat Commun (2013) 4:2771. doi: 10.1038/ncomms3771 24264146 PMC3868224

[17] Johnson MR Behmoaras J Bottolo L Krishnan ML Pernhorst K Santoscoy P. Systems genetics identifies sestrin 3 as a regulator of a proconvulsant gene network in human epileptic hippocampus. Nat Commun (2015) 6:6031. doi: 10.1038/ncomms7031 25615886 PMC4627576

[18] Rawat C Kutum R Kukal S Srivastava A Dahiya UR Kushwaha S. Downregulation of peripheral PTGS2/COX-2 in response to valproate treatment in patients with epilepsy. Sci Rep (2020) 10(1):2546. doi: 10.1038/s41598-020-59259-x 32054883 PMC7018850

[19] Wang ZB Qu J Yang ZY Liu DY Jiang SL Zhang Y. Integrated analysis of expression profile and potential pathogenic mechanism of temporal lobe epilepsy with hippocampal sclerosis. Front Neurosci (2022) 16:892022. doi: 10.3389/fnins.2022.892022 35784838 PMC9243442

[20] Kumar P Lim A Hazirah SN Chua C Ngoh A Poh SL. Single-cell transcriptomics and surface epitope detection in human brain epileptic lesions identifies pro-inflammatory signaling. Nat Neurosci (2022) 25(7):956–66. doi: 10.1038/s41593-022-01095-5 PMC9276529 35739273

[21] Fitzgibbons TP Edwards Y Shaw P Iskandar A Ahmed M Bote J. Activation of inflammatory and pro-thrombotic pathways in acute stress cardiomyopathy. Front Cardiovasc Med (2017) 4:49. doi: 10.3389/fcvm.2017.00049 28824923 PMC5541033

[22] Goh W Wang W Wong L. Why batch effects matter in omics data, and how to avoid them. Trends Biotechnol (2017) 35(6):498–507. doi: 10.1016/j.tibtech.2017.02.012 28351613

[23] Ritchie ME Phipson B Wu D Hu Y Law CW Shi W. Limma powers differential expression analyses for RNA-sequencing and microarray studies. Nucleic Acids Res (2015) 43(7):e47. doi: 10.1093/nar/gkv007 25605792 PMC4402510

[24] Xiao S Zhou Y Liu A Wu Q Hu Y Liu J. Uncovering potential novel biomarkers and immune infiltration characteristics in persistent atrial fibrillation using integrated bioinformatics analysis. Math Biosci Eng MBE (2021) 18(4):4696–712. doi: 10.3934/mbe.2021238 34198460

[25] Wu T Hu E Xu S Chen M Guo P Dai Z. clusterProfiler 4.0: A universal enrichment tool for interpreting omics data. Innovation (Cambridge (Mass.)) (2021) 2(3):100141. doi: 10.1016/j.xinn.2021.100141 34557778 PMC8454663

[26] Wang F Yuan C Wu HZ Liu B Yang YF. Bioinformatics, molecular docking and experiments *In vitro* analyze the prognostic value of CXC chemokines in breast cancer. Front Oncol (2021) 11:665080. doi: 10.3389/fonc.2021.665080 34123826 PMC8189319

[27] Shannon P Markiel A Ozier O Baliga NS Wang JT Ramage D. Cytoscape: A software environment for integrated models of biomolecular interaction networks. Genome Res (2003) 13(11):2498–504. doi: 10.1101/gr.1239303 PMC403769 14597658

[28] Chen S Yang D Lei C Li Y Sun X Chen M. Identification of crucial genes in abdominal aortic aneurysm by WGCNA. PeerJ (2019) 7:e7873. doi: 10.7717/peerj.7873 31608184 PMC6788446

[29] Chin CH Chen SH Wu HH Ho CW Ko MT Lin CY. cytoHubba: Identifying hub objects and sub-networks from complex interactome. BMC Syst Biol (2014) 8 Suppl 4(Suppl 4):S11. doi: 10.1186/1752-0509-8-S4-S11 25521941 PMC4290687

[30] Franz M Rodriguez H Lopes C Zuberi K Montojo J Bader GD. GeneMANIA update 2018. Nucleic Acids Res (2018) 46(W1):W60–4. doi: 10.1093/nar/gky311 PMC6030815 29912392

[31] Lang M Binder M Richter J Schratz P Pfisterer F Coors S. mlr3: A modern object-oriented machine learning framework in r. J Open Source Software (2019) 4(44), 1903. doi: 10.21105/joss.01903

[32] Yang L Wu H Jin X Zheng P Hu S Xu X. Study of cardiovascular disease prediction model based on random forest in eastern China. Sci Rep (2020) 10(1):5245. doi: 10.1038/s41598-020-62133-5 32251324 PMC7090086

[33] Ogunleye A Wang QG. XGBoost model for chronic kidney disease diagnosis. IEEE/ACM Trans Comput Biol Bioinf (2020) 17(6):2131–40. doi: 10.1109/TCBB.2019.2911071 30998478

[34] Smedley D Haider S Durinck S Pandini L Provero P Allen J. The BioMart community portal: an innovative alternative to large, centralized data repositories. Nucleic Acids Res (2015) 43(W1):W589–98. doi: 10.1093/nar/gkv350 PMC4489294 25897122

[35] Kawada JI Takeuchi S Imai H Okumura T Horiba K Suzuki T. Immune cell infiltration landscapes in pediatric acute myocarditis analyzed by CIBERSORT. J Cardiol (2021) 77(2):174–8. doi: 10.1016/j.jjcc.2020.08.004 32891480

[36] Wang X Wen D You C Ma L. Identification of the key immune-related genes in aneurysmal subarachnoid hemorrhage. Front Mol Neurosci (2022) 15:931753. doi: 10.3389/fnmol.2022.931753 36172261 PMC9511034

[37] Hao Y Hao S Andersen-Nissen E Mauck WM 3rd Zheng S Butler A. Integrated analysis of multimodal single-cell data. Cell (2021) 184(13):3573–3587.e29. doi: 10.1016/j.cell.2021.04.048 34062119 PMC8238499

[38] Zhang X Lan Y Xu J Quan F Zhao E Deng C. CellMarker: a manually curated resource of cell markers in human and mouse. Nucleic Acids Res (2019) 47(D1):D721–8. doi: 10.1093/nar/gky900 PMC6323899 30289549

[39] Aran D Looney AP Liu L Wu E Fong V Hsu A. Reference-based analysis of lung single-cell sequencing reveals a transitional profibrotic macrophage. Nat Immunol (2019) 20(2):163–72. doi: 10.1038/s41590-018-0276-y PMC6340744 30643263

[40] Stoeckius M Hafemeister C Stephenson W Houck-Loomis B Chattopadhyay PK Swerdlow H. Simultaneous epitope and transcriptome measurement in single cells. Nat Methods (2017) 14(9):865–8. doi: 10.1038/nmeth.4380 PMC5669064 28759029

[41] Wu Y Yang S Ma J Chen Z Song G Rao D. Spatiotemporal immune landscape of colorectal cancer liver metastasis at single-cell level. Cancer Discovery (2022) 12(1):134–53. doi: 10.1158/2159-8290.CD-21-0316 34417225

[42] Samuels MA. The brain-heart connection. Circulation (2007) 116(1):77–84. doi: 10.1161/CIRCULATIONAHA.106.678995 17606855

[43] Gilad Y Mizrahi-Man O. A reanalysis of mouse ENCODE comparative gene expression data. F1000Research (2015) 4:121. doi: 10.12688/f1000research.6536.1 26236466 PMC4516019

[44] Fortunato F Giugno A Sammarra I Labate A Gambardella A. Epilepsy, immunity and neuropsychiatric disorders. Curr Neuropharmacol (2022). doi: 10.2174/1570159X20666220706094651 PMC10514543 35794773

[45] Merelli A Repetto M Lazarowski A Auzmendi J. Hypoxia, oxidative stress, and inflammation: Three faces of neurodegenerative diseases. J Alzheimer's Dis JAD (2021) 82(s1):S109–26. doi: 10.3233/JAD-201074 33325385

[46] Pei Q Mbabazi N Zou L Zhang J Yin H Li B. Mechanisms of myocardial stunning in stress-induced cardiomyopathy. Cardiovasc Innov Appl (2022). doi: 10.15212/CVIA.2022.0010

[47] Xin P Xu X Deng C Liu S Wang Y Zhou X. The role of JAK/STAT signaling pathway and its inhibitors in diseases. Int Immunopharmacol (2020) 80:106210. doi: 10.1016/j.intimp.2020.106210 31972425

[48] Merelli A Ramos AJ Lazarowski A Auzmendi J. Convulsive stress mimics brain hypoxia and promotes the p-glycoprotein (P-gp) and erythropoietin receptor overexpression. recombinant human erythropoietin effect on p-gp activity. Front Neurosci (2019) 13:750. doi: 10.3389/fnins.2019.00750 31379495 PMC6652211

[49] Yang Z Kirton HM Al-Owais M Thireau J Richard S Peers C. Epac2-Rap1 signaling regulates reactive oxygen species production and susceptibility to cardiac arrhythmias. Antioxid Redox Signaling (2017) 27(3):117–32. doi: 10.1089/ars.2015.6485 PMC5510674 27649969

[50] Santos RAS Sampaio WO Alzamora AC Motta-Santos D Alenina N Bader M. The ACE2/Angiotensin-(1-7)/MAS axis of the renin-angiotensin system: Focus on angiotensin-(1-7). Physiol Rev (2018) 98(1):505–53. doi: 10.1152/physrev.00023.2016 PMC7203574 29351514

[51] Papaconstantinou J. The role of signaling pathways of inflammation and oxidative stress in development of senescence and aging phenotypes in cardiovascular disease. Cells (2019) 8(11):1383. doi: 10.3390/cells8111383 31689891 PMC6912541

[52] Krem MM Di Cera E. Molecular markers of serine protease evolution. EMBO J (2001) 20(12):3036–45. doi: 10.1093/emboj/20.12.3036 PMC150214 11406580

[53] Feyissa AM Hasan TF Meschia JF. Stroke-related epilepsy. Eur J Neurol (2019) 26(1):18–e3. doi: 10.1111/ene.13813 30320425

[54] Barnes SE Zera KA Ivison GT Buckwalter MS Engleman EG. Brain profiling in murine colitis and human epilepsy reveals neutrophils and TNFα as mediators of neuronal hyperexcitability. J Neuroinflamm (2021) 18(1):199. doi: 10.1186/s12974-021-02262-4 PMC8436533 34511110

[55] Zhang D Hu W Tu H Hackfort BT Duan B Xiong W. Macrophage depletion in stellate ganglia alleviates cardiac sympathetic overactivation and ventricular arrhythmogenesis by attenuating neuroinflammation in heart failure. Basic Res Cardiol (2021) 116(1):28. doi: 10.1007/s00395-021-00871-x 33884509 PMC8060235

[56] Ciutac AM Dawson D. The role of inflammation in stress cardiomyopathy. Trends Cardiovasc Med (2021) 31(4):225–30. doi: 10.1016/j.tcm.2020.03.005 32276825

[57] Scally C Abbas H Ahearn T Srinivasan J Mezincescu A Rudd A. Myocardial and systemic inflammation in acute stress-induced (Takotsubo) cardiomyopathy. Circulation (2019) 139(13):1581–92. doi: 10.1161/CIRCULATIONAHA.118.037975 PMC6438459 30586731

[58] Tian W Zhou J Chen M Qiu L Li Y Zhang W. Bioinformatics analysis of the role of aldolase a in tumor prognosis and immunity. Sci Rep (2022) 12(1):11632. doi: 10.1038/s41598-022-15866-4 35804089 PMC9270404

[59] Linnerbauer M Wheeler MA Quintana FJ. Astrocyte crosstalk in CNS inflammation. Neuron (2020) 108(4):608–22. doi: 10.1016/j.neuron.2020.08.012 PMC7704785 32898475

[60] Hu YH Liu J Lu J Wang PX Chen JX Guo Y. sFRP1 protects H9c2 cardiac myoblasts from doxorubicin-induced apoptosis by inhibiting the Wnt/PCP-JNK pathway. Acta Pharmacol Sin (2020) 41(9):1150–7. doi: 10.1038/s41401-020-0364-z PMC7608092 32238888

[61] Diao L Yu H Li H Hu Y Li M He Q. LncRNA UCA1 alleviates aberrant hippocampal neurogenesis through regulating miR-375/SFRP1-mediated WNT/β-catenin pathway in kainic acid-induced epilepsy. Acta Biochim Polonica (2021) 68(2):159–67. doi: 10.18388/abp.2020_5448 33829718

[62] Yin H Favreau-Lessard AJ deKay JT Herrmann YR Robich MP Koza RA. Protective role of ErbB3 signaling in myeloid cells during adaptation to cardiac pressure overload. J Mol Cell Cardiol (2021) 152:1–16. doi: 10.1016/j.yjmcc.2020.11.009 33259856 PMC7981250

[63] Zhu J Zhu Z Ren Y Dong Y Li Y Yang X. Role of the Nrdp1 in brain injury induced by chronic intermittent hypoxia in rats *via* regulating the protein levels of ErbB3. Neurotoxicity Res (2020) 38(1):124–32. doi: 10.1007/s12640-020-00195-z 32200526

[64] Hadchouel A Durrmeyer X Bouzigon E Incitti R Huusko J Jarreau PH. Identification of SPOCK2 as a susceptibility gene for bronchopulmonary dysplasia. Am J Respir Crit Care Med (2011) 184(10):1164–70. doi: 10.1164/rccm.201103-0548OC PMC4826668 21836138

[65] Hadchouel A Franco-Montoya ML Guerin S Do Cruzeiro M Lhuillier M Ribeiro Baptista B. Overexpression of Spock2 in mice leads to altered lung alveolar development and worsens lesions induced by hyperoxia. Am J Physiol Lung Cell Mol Physiol (2020) 319(1):L71–81. doi: 10.1152/ajplung.00191.2019 32374670

[66] Wu T Dejanovic B Gandham VD Gogineni A Edmonds R Schauer S. Complement C3 is activated in human AD brain and is required for neurodegeneration in mouse models of amyloidosis and tauopathy. Cell Rep (2019) 28(8):2111–2123.e6. doi: 10.1016/j.celrep.2019.07.060 31433986

[67] Yuan K Ye J Liu Z Ren Y He W Xu J. Complement C3 overexpression activates JAK2/STAT3 pathway and correlates with gastric cancer progression. J Exp Clin Cancer Res CR (2020) 39(1):9. doi: 10.1186/s13046-019-1514-3 31928530 PMC6956509

[68] Harland M Torres S Liu J Wang X. Neuronal mitochondria modulation of LPS-induced neuroinflammation. J Neurosci (2020) 40(8):1756–65. doi: 10.1523/JNEUROSCI.2324-19.2020 PMC7046320 31937559

[69] Wang M Zhong H Zhang X Huang X Wang J Li Z. EGCG promotes PRKCA expression to alleviate LPS-induced acute lung injury and inflammatory response. Sci Rep (2021) 11(1):11014. doi: 10.1038/s41598-021-90398-x 34040072 PMC8154949

