# Supplementary figures and images for: Single-cell sequencing combined with machine learning reveals the mechanism of interaction between epilepsy and stress cardiomyopathy
Source: Front Immunol. 2023 Jan 27;14:1078731. doi: 10.3389/fimmu.2023.1078731 (PMC9911815; doi:10.3389/fimmu.2023.1078731)

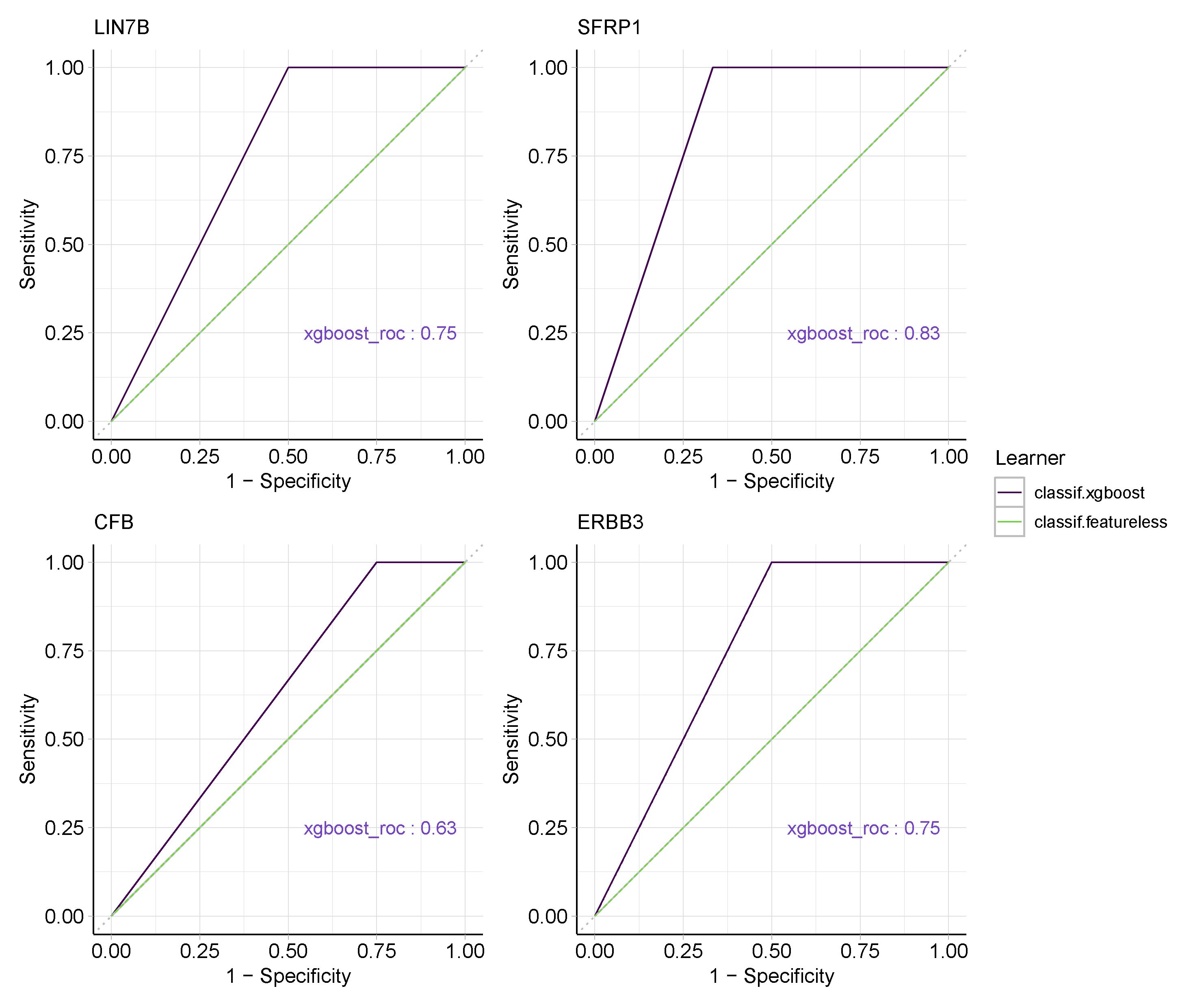

Supplement: Supplementary file 1 [file Image_1.jpeg]
